# Implementation of cognitive assessment and follow‐ups in people complaining of cognitive problems over the age of 50, in community pharmacies by using the Neurocognitive Frailty Index: A pilot study

**DOI:** 10.1002/alz70858_103813

**Published:** 2025-12-25

**Authors:** Sarah Pakzad, Paul Bourque, Daniel Saucier

**Affiliations:** ^1^ Université de Moncton, Moncton, NB, Canada; ^2^ University of Moncton, Moncton, NB, Canada

## Abstract

**Background:**

Pharmacists are an integral and accessible part of the community that play an evolving role in Canadian primary health care (Raiche et al., 2020), while integrating technology in the cognitive assessment process could help with dementia care and management (Astell et al., 2019). The aim of the current ongoing pilot study is to examine how pharmacists’ utilization of the digital version of the Neurocognitive Frailty Index (NFI, Pakzad et al., 2017) can improve timely identification of cognitive disorder and contribute to the quality of treatment and care in patients with cognitive complaints, over time.

**Method:**

Pharmacies across New Brunswick, Canada were invited to participate in the current study as to receive training to offer the NFI assessment tool to their clients aged 50 years and over with repeat assessments spaced 3‐12 months apart. Longitudinal analyses will allow for proper follow‐up of study participants as to ascertain the pertinence of NFI in tracking cognitive decline, as well as noting the professional healthcare decisions made by pharmacists based on the personalized report generated by the NFI.

**Result:**

A total of 9 pharmacists have been recruited and trained on the administration of NFI. The first wave of NFI assessments began in November 2024 with 12 individuals tested thus far, as well as receiving a follow‐up by their pharmacists regarding scheduling a follow‐up NFI evaluation, referral to a primary care provider, having their prescriptions reviewed and/or adjusted, recommending lifestyle changes and/or offering any cognitive pharmaceutical services if available within their respective pharmacies.

**Conclusion:**

Recruitment and training of additional pharmacists is ongoing, while already active pharmacists have signaled continued interest to offer the NFI to their clients at their respective pharmacies. By the end of this pilot study, the results will help inform whether the implementation and use of the NFI by pharmacists as a companion tool will positively impact the management and outcome of those individuals aged 50 years and over with cognitive impairment.

